# The effect of freeze–thaw and storage on African swine fever virus detection in environmental samples

**DOI:** 10.3389/fvets.2025.1570575

**Published:** 2025-04-01

**Authors:** Taeyong Kwon, Jordan T. Gebhardt, Eu Lim Lyoo, Natasha N. Gaudreault, Jessie D. Trujillo, Juergen A. Richt

**Affiliations:** Department of Diagnostic Medicine/Pathobiology, College of Veterinary Medicine, Kansas State University, Manhattan, KS, United States

**Keywords:** African swine fever, ASFV, environmental sample, freeze–thaw, fomite, surface, storage

## Abstract

African swine fever is a devastating viral disease of swine causing up to 100% mortality and significant impacts to the swine industry. The causative agent, African swine fever virus (ASFV), is a large, enveloped virus containing a linear, double-stranded DNA genome with 170–190 kb in length. Since its introduction into the Caucasus region in 2007, the genotype II ASFV has continued to spread to Europe, Asia, and Caribbean countries. Early detection is crucial to prevent and control ASF outbreaks for biosecurity purposes, and environmental samples can be used to evaluate the level of biosecurity. Therefore, we evaluated the effect of freeze–thaw cycles and storage at 4°C and room temperature (RT) on ASFV DNA detection in environmental samples. ASFV DNA was stable in environmental samples with no organic contaminants after freeze–thaw and incubation at 4°C and RT. However, incubation at RT negatively affects ASFV detection in swine feces and feed dust samples that were collected using premoistened gauze. There were significant reductions in ASFV detection in environmental samples in the presence of soil and organic mixture after freeze–thaw and incubation at 4°C and RT. These results provide novel insights on the appropriate storage of environmental samples for ASFV detection and contribute to the control and prevention of ASF outbreaks and new introductions.

## 1 Introduction

African swine fever (ASF) is one of the most devastating diseases in the swine industry due to its significant impact on swine health and socioeconomic consequences in affected countries. ASF is a severe, systemic hemorrhagic disease of pigs that produces a wide range of clinical signs and lesions, caused by the African swine fever virus (ASFV). ASFV is a large enveloped, double-stranded DNA virus and belongs to the family *Asfarviridae*. The virus has strict host specificity capable of infecting animal species in the family *Suidae*, including domestic pigs and wild boars. The infection in African wild pigs, such as warthogs and bushpigs, is asymptomatic but plays a critical role in ASFV transmission and persistence in Sub-Saharan Africa (1). The recent epidemic is associated with a genotype II virus and originated from an intercontinental transmission from Eastern Africa to Georgia in 2007 (2). The virus subsequently spread to Russia as well as eastern and central European countries. It is assumed that infected wild boar played a critical role in ASFV transmission in these areas (3). In 2018, ASF outbreaks were first reported in China and rapidly spread to nearby Asian countries; human activities were the main contributors to the spread of the virus over long distances (4). The capability of ASFV to cross intercontinental, geographical barriers was indeed supported by ASF outbreaks in the Dominican Republic in 2021 (5).

ASFV transmission occurs through direct contact with infected pigs, wild boar, and soft ticks as well as via indirect routes such as contaminated fomites or pork products. The virus exhibits high stability in pork and pork products, thus contaminated pork and pork products are one of the greatest sources for indirect ASFV transmission (6). In addition, it has been well-documented that the virus is highly resistant in the environment on a wide variety of surface types. The virus retains infectivity for a few weeks to several months in a variety of environmental substrates and surfaces, suggesting the potential role of environmental contamination in ASFV transmission (7–9). For this reason, environmental sampling procedures are useful tools to evaluate the level of biosecurity in pig production system in order to identify shortcomings of biosecurity practices and to determine where enhanced practices should be incorporated to prevent transmission of ASFV or other pathogens. In an ASFV-affected country, a recent investigation utilized environmental sampling techniques in the feed supply chain and found that 0.7% of environmental samples were positive for ASFV; the results were used to implement additional biosecurity procedures (10). Previously, we developed an environmental sampling technique and associated processing methods for detecting ASFV DNA with high sensitivity (11, 12). However, there remained a knowledge gap regarding the storage of samples collected from environmental sampling procedures since scientifically-supported best practices are not currently available. The aim of the present study was to determine the effect of sample storage and handling procedures on detection of ASFV DNA in environmental samples from surfaces contaminated with ASFV and a range of organic materials.

## 2 Materials and methods

### 2.1 Ethics statement and virus

All experiments were approved under the Kansas State University (KSU) Institutional Biosafety Committee (IBC, Protocol #1600) and performed in a biosafety level-3 laboratory in the Biosecurity Research Institute at KSU. Whole blood was collected from Georgia07-infected pigs from a separate animal study and stored at – 80°C until the experiment was conducted. The virus titer was 3.68 × 10^8^ TCID_50_/mL.

### 2.2 Surface contamination, sample collection and processing

Surface contamination was performed as previously described (11, 12). Briefly, 100 μL ASFV-infected blood were mixed with (1) 5 mL of phosphate buffered saline (PBS) or 2.5 g of each organic contaminant and 2.5 mL of phosphate buffered saline (PBS): (2) soil, (3) swine feces collected from finishing pigs, (4) feed dust collected from feed mill, and (5) mixture of soil, swine feces, and feed dust. The mixture was inoculated on a 10 × 10 cm stainless steel surface and dried for 30 min at RT in a biosafety cabinet. The surface was swabbed using either pre-moisten cotton gauze (Dynarex Corporation, Orangeburg, NY, United States) with 5 mL of PBS or a sponge stick (Cat. #SSL100, 3 M, MN, United States) pre-moistened with 10 mL DNA/RNA shield (Zymo Research, Irvine, CA). The cotton gauze was placed in a 50 mL conical tube, and the sponge stick was returned to a plastic bag. Positive control 100 μL ASFV-infected blood in 15 or 20 mL of PBS. These samples were subjected to three different storage conditions: (1) freezing at – 80°C and thawing 0X, 1X, 2X, or 3X, (2) storage at 4°C for 0, 1, 3, or 7 days, and (3) storage at room temperature (RT) for 0, 1, 3, or 7 days. After storage, the gauze samples were processed by adding 5 mL of PBS, vortexing, and incubating for 5 min at RT. The sponge stick was massaged and incubated for 5 min. For the sponge stick samples from feed dust and mix-contaminated surfaces, an additional 5 mL of PBS was added for facilitating the elution step. The supernatant was transferred into a microtube and centrifuged at 700 × g for 5 min. The clarified supernatant was mixed with the equal volume of AL lysis buffer (Qiagen, Germantown, MD, United States) and stored until further analysis. Each treatment combination was replicated three times.

### 2.3 DNA extraction and quantitative PCR

Viral DNA extraction was performed using an automated magnetic bead-based extraction system as previously described (13). Briefly, the AL lysate was heat-inactivated at 70°C for 10 min. A total of 200 μL of AL lysate and 200 μL of isopropanol was added into an extraction plate that was pre-filled with extraction reagents, and extraction was performed in the automated extractor. The eluted DNA was mixed with p72-specific primer and probe set in PCR mastermix. PCR was performed in duplicate using the CFX 96 PCR machine. The Cq value was converted to copy numbers/mL using the standard curve generated from serial dilutions of the known concentration of the plasmid containing the target p72 sequence.

### 2.4 Statistical analysis

Data were analyzed by a linear model fit using the GLIMMIX function in SAS (version 9.4, Cary, NC) with the response variable for all models being ASFV p72 Log10 copy number/mL. For analysis of storage time at 4°C or RT, sampling device treatment with respective organic matter contamination level, day of storage, and the associated interaction were fit in the model as fixed effects. A similar statistical model was fit to assess the effect of freeze–thaw storage with sampling device treatment with respective organic matter contamination level, number of freeze–thaw cycles, and the associated interaction as fixed effects. In both models, linear and quadratic contrast statements were incorporated to determine the change in ASFV DNA detection by freeze–thaw cycle and over time, respectively. The SLICE function was used to perform an *F*-test within each sampling device treatment to test the effect of day or number of freeze–thaw cycles. Visual assessment of studentized residual plots was performed to evaluate model assumptions which appeared to be reasonably met. A Tukey multiple comparison adjustment was used to control Type I error rate, and results were considered significant at *p* < 0.05.

## 3 Results

To determine the effect of freeze–thaw cycles on ASFV DNA stability, the environmental samples were subjected to freeze–thaw cycles 0X, 1X, 2X, or 3X. There was no significant reduction of ASFV DNA detection in all environmental samples that were collected using a premoistened gauze (Table 1; *p* > 0.05). In parallel, there was no effect (*p* > 0.05) of freeze–thaw on ASFV DNA detection in samples collected from surfaces with no organic material contamination, feces, feed dust, and other organic mixture samples that were collected using the sponge stick with DNA/RNA shield. In contrast, ASFV DNA detection was significantly reduced (*p* < 0.05 and 0.4 log reduction after incubation for 7 days) after a 3X freeze–thaw cycle compared to the sample with 0X freeze–thaw cycles in samples that were collected using a sponge stick with DNA/RNA shield on surfaces with soil contamination.

**Table 1:** Effect of freeze–thaw cycles on detection of ASFV p72 log_10_ copy number/mL.

	Freeze/thaw cycles				*P* =		
	0	1	2	3	Treatment	Linear	Quadratic
Treatment							
PC, 15 mL	6.34	6.42	6.38	6.23	0.563	0.412	0.244
PC, 20 mL	6.39	6.31	6.38	6.22	0.588	0.323	0.640
Gauze							
Non-organic	5.96	5.78	6.07	5.86	0.188	0.963	0.877
Soil	4.86	4.81	4.65	4.92	0.264	0.982	0.118
Swine feces	4.94	4.91	5.07	5.14	0.301	0.085	0.633
Feed dust	5.60	5.75	5.73	5.78	0.544	0.225	0.608
Organic mixture	4.20	4.08	4.22	4.10	0.675	0.724	0.992
Sponge							
Non-organic	6.19	6.34	6.33	6.20	0.570	0.920	0.160
Soil	6.13^a^	5.87^ab^	6.00^ab^	5.69^b^	0.014	0.007	0.790
Swine feces	6.44	6.38	6.29	6.29	0.641	0.225	0.737
Feed dust	6.51	6.42	6.43	6.62	0.442	0.419	0.158
Organic mixture	6.02	6.00	6.11	5.84	0.271	0.350	0.206

^1SEM = 0.098. Treatment × freeze/thaw cycle, P = 0.285. Main effect of freeze/thaw cycle, P = 0.310. Main effect of sample type, P < 0.0001. Means within row lacking common superscript differ, p < 0.05. PC = positive control; non-organic = PBS.^

Next, we investigated the effect of storage at 4°C on ASFV DNA detection. Samples were incubated at 4°C for 0, 1, 3, and 7 days after sample collection. There was a significant interaction between treatment and the time (days) of storage (Table 2; *p* < 0.0001). The effect of storage time was dependent upon the sampling device and level of surface organic matter contamination. In non-organic, swine feces and feed dust samples using either sampling device, no ASFV DNA degradation was observed. ASFV DNA concentrations significantly decreased over time for samples collected from surfaces contaminated with soil using the premoistened gauze sampling device (Linear, *p* < 0.0001 and 1.2 log reduction after incubation for 7 days). In addition, ASFV DNA detection was significantly reduced in samples collected from surfaces contaminated with the organic mixture using the sponge stick with DNA/RNA shield, with the greatest reduction in detection occurring within 1 day of incubation at 4°C with no subsequent reduction when stored beyond 1 day (Quadratic, *p* < 0.0001 and 0.5 log reduction after incubation for 7 days). There was no evidence of a difference in ASFV DNA detection based on day of storage for any of the other combinations of sampling device and organic matter contamination level (*p* > 0.05).

**Table 2:** Effect of storage at 4°C on detection of ASFV p72 log_10_ copy number/mL^1^.

	Days of storage				*P* =		
	0	1	3	7	Treatment	Linear	Quadratic
Treatment							
PC, 15 mL	6.44	6.47	6.26	6.40	0.538	0.675	0.265
PC, 20 mL	6.43	6.33	6.22	6.41	0.476	0.935	0.117
Gauze							
Non-organic	6.12	6.10	5.95	5.92	0.408	0.122	0.553
Soil	5.15^a^	4.56^b^	4.43^b^	3.90^c^	< 0.0001	< 0.0001	0.084
Swine feces	5.84	6.09	6.09	6.01	0.300	0.557	0.139
Feed dust	5.86	5.79	5.68	5.70	0.584	0.287	0.372
Organic mixture	5.07	4.71	4.78	4.80	0.084	0.297	0.103
Sponge							
Non-organic	6.49	6.19	6.43	6.30	0.194	0.587	0.899
Soil	6.06	6.08	5.92	6.04	0.701	0.815	0.353
Swine feces	6.54	6.19	6.33	6.47	0.092	0.619	0.104
Feed dust	6.31	6.23	6.37	6.32	0.838	0.709	0.758
Organic mixture	6.38^a^	5.82^b^	5.60^b^	5.86^b^	< 0.0001	0.014	< 0.0001

1 SEM = 0.106. Treatment × day, *P* < 0.0001. Main effect of day, *P* < 0.0001. Main effect of sample type, *P* < 0.0001.

Means within row lacking common superscript differ, *P* < 0.05. PC = positive control; non-organic = PBS.

Lastly, the effect of storage at RT on ASFV DNA detection was studied in environmental samples. Similar to storage at 4°C, there was a treatment × day effect (Table 3; *p* < 0.0001). ASFV DNA was stable over time in samples collected from surfaces with no organic matter contamination using either sampling device, but significant reductions of ASFV DNA were observed in soil, feces, and feed dust samples collected with the premoistened gauze (*p* ≤ 0.043 and 1.5, 0.2, and 1.2 log reduction, respectively, after incubation for 7 days). Using the sponge stick with DNA/RNA shield samples, ASFV DNA was stable in swine feces and feed dust, however detection of ASFV DNA gradually decreased in the presence of soil and organic mixture (Quadratic, *p* ≤ 0.003 and 0.6 log reduction, respectively, after incubation for 7 days). There was no evidence of a reduction in ASFV DNA when environmental samples were collected using the sponge stick with DNA/RNA shield from surfaces contaminated with swine feces or feed dust (*p* > 0.05).

**Table 3:** Effect of storage at room temperature on detection of ASFV p72 log_10_ copy number/mL^1^.

	Days of storage				*P* =		
	0	1	3	7	Treatment	Linear	Quadratic
Treatment							
PC, 15 mL	6.15	6.00	6.06	6.10	0.762	0.987	0.517
PC, 20 mL	6.08	6.04	6.02	6.28	0.261	0.097	0.266
Gauze							
Non-organic	6.18	6.06	6.05	6.15	0.748	0.960	0.314
Soil	4.96^a^	4.37^b^	3.28^c^	3.38^c^	< 0.0001	< 0.0001	< 0.0001
Swine feces	5.16	5.07	5.00	4.87	0.233	0.043	0.772
Feed dust	5.84^a^	5.18^b^	4.87^bc^	4.64^c^	< 0.0001	< 0.0001	0.0001
Organic mixture	4.37	4.40	4.39	4.39	0.998	0.971	0.916
Sponge							
Non-organic	6.52	6.24	6.24	6.34	0.163	0.477	0.072
Soil	6.28^a^	5.93^ab^	5.52^c^	5.62^bc^	< 0.0001	< 0.0001	0.0002
Swine feces	6.58	6.29	6.30	6.35	0.139	0.300	0.102
Feed dust	6.52	6.23	6.37	6.28	0.219	0.330	0.531
Organic mixture	6.25^a^	5.91^ab^	5.66^b^	5.72^b^	0.0003	0.001	0.003

1 SEM = 0.101. Treatment × day, *P* < 0.0001. Main effect of day, *P* < 0.0001. Main effect of sample type, *P* < 0.0001.

Means within row lacking common superscript differ, *P* < 0.05. PC = positive control; non-organic = PBS.

## 4 Discussion

Due to the lack of an effective and safe vaccine to control and prevent ASFV infection, current approaches rely on enhanced biosecurity to prevent the introduction of ASFV into ASFV-free areas and to confine and destroy the source of infections. Early detection is one of the most important factors to determine successful implementation of biosecurity. The detection of ASFV DNA is a gold standard for agent identification, and most diagnostic laboratories utilize automated DNA extraction and real-time PCR for ASFV detection (14). In addition, novel assays, such as point-of-care detection, have been developed for fast turnaround time and increased sensitivity (15). Clinical samples from pigs or wild boar are the most popular sample type for detecting ASFV DNA, thus these types of samples, such as blood and tissue, are validated for molecular diagnostics. In contrast, a few manuscripts attempted to validate and improve the use of environmental samples for ASFV detection (11, 12). The successful ASFV DNA detection in environmental samples is affected by several factors: choice of sampling devices, appropriate sample collection from the targeted area, rapid transportation of samples to the diagnostic laboratory, and the validated assays for ASFV DNA detection. In modern swine production systems, samples are usually shipped to diagnostic laboratory in a few days. Although overnight shipping of samples is common in the United States, some countries experience delayed sample transportation, and subsequently diagnostic sensitivity could be greatly compromised. In particular, most environmental samples contain a variety of organic contaminants, in which different types of proteinases, DNases, and RNases can potentially degrade nucleic acids and/or inhibit subsequent molecular analysis. Therefore, this study aimed to evaluate the effect of sample storage conditions on ASFV DNA detection in environmental samples.

The efforts of the swine industry in the past focused on preserving the integrity of RNA molecules of RNA viruses in diagnostic specimens because of the economic impact of endemic RNA viruses, such as porcine reproductive and respiratory syndrome virus (PRRSV) (16). A previous study showed that PRRSV detection was maintained for up to 168 h in serum that was stored at 4, 10 or 20°C; however, incubation at 30°C had a negative effect on PRRSV RNA stability (17). In contrast, PRRSV RNA detection decreased in oral fluid and fecal samples over time at 4, 10, 20, or 30°C. The reduced detection of porcine epidemic diarrhea virus (PEDV) and transmissible gastroenteritis virus (TGEV) RNA in fecal samples was observed after storage at 4, 21, 36, and 45°C (18). DNA in general is more stable than RNA, however, prolonged storage of ASFV-infected tissues at 4 and 23°C led to reduction of ASFV DNA detection (8). Our data supports the high stability of ASFV DNA in environmental samples with no contamination with organic materials such as feed dust, fecal material, or soil. However, we observed significant reduction in the stability of ASFV DNA in environmental samples with organic contaminants, in particular in the presence of soil, suggesting that ASFV DNA stability is dependent on the type of organic contaminant. Bacteria and fungi play an important role in building the microenvironment of soils and produce a variety of extracellular DNases which could affect the stability of DNA viruses. The optimal temperature of soil DNase activity is between 30 to 40°C, but DNase activity can be sufficient for DNA degradation even at 4°C (19, 20). It could be plausible that soil DNases in environmental samples contaminated with soil could result in ASFV DNA degradation in this study. In addition, the pH level may also affect ASFV DNA stability. Soil could have an acidic or alkaline pH level which may also affect DNAse activity (21). Furthermore, we also observed reduced ASFV DNA concentrations in some samples collected with the sponge stick with DNA/RNA shield after freeze–thaw cycles and incubation at 4°C and RT. The role of DNA/RNA shield in preserving nucleic acids has been well-documented, and our study confirmed better sensitivity with the sponge stick plus DNA/RNA shield than the gauze plus PBS. Importantly, the volume of DNA/RNA shield (10 mL) may not be sufficient to preserve ASFV DNA in environmental samples containing excessive amounts of organic contaminants (2.5 g) as the manufacturer’s instruction indicates an optimal ratio of 10% (v/v and w/v), since the present study stimulated the field situation where organic matters are heavily contaminating environment samples.

Environmental samples have been used to monitor disease status and the distribution of infectious agents in swine herds after a disease outbreak. For example, following a PRRS outbreak, viral RNA remained detectable up to 14 weeks post-outbreak in environmental wipes and the sensitivity of environmental samples was similar to blood samples (22). In addition, environmental sampling revealed the extensive contamination and distribution of PRRSV RNA on contaminated farms, such as exhaust fan cones, door knobs, anteroom floors, mortality carts/sleds (23). Similarly, long-term persistence and extensive contamination of PEDV RNA was found after an outbreak and the results of environmental samples were useful to implement a modified biosecurity protocol (24). In contrast to endemic viruses, it is impossible to monitor the disease status in ASFV-affected farms over time because the affected herd is readily culled for control purposes. Rather, the environmental samples can be used to evaluate the level of cleaning and disinfecting process for the re-introduction of animals after the ASF outbreak. In addition, they are valuable sample types to detect ASFV on equipment and transportation vehicles in affected areas, in order to reduce ASFV transmission between farms. In this scenario, the environmental samples should be properly collected and immediately shipped to the diagnostic laboratory at 4°C. If not possible, our data support and recommend freezing the environmental samples before shipping. We found when using different sampling devices with differing organic contamination levels, there were minimal reductions in ASFV DNA concentrations; even after 3 freeze–thaw cycles, the amount of ASFV DNA recovered still is likely to generate positive PCR results.

One of limitations of our study is the high virus titer for surface contamination, approximately 3.7 × 10^7^ TCID_50_ per sample type. This amount of virus is certainly present in the blood of infected animals. However, in reality, ASFV DNA is present at a variable level in environmental samples and therefore, ASFV DNA decay on various surfaces may be more variable, compared to that of high level of contamination used in this study. Secondly, we tested the viral DNA stability in certain types of organic contaminants on a single surface type, stainless steel. Others surfaces such as cement, plastic and cardboard present on farms were not tested. Therefore, further exploration using a variety of infectious loads, organic contaminants and surfaces would provide additional understanding of viral DNA decay in environmental samples. Lastly, due to the limitations at the BSL-3 facility, the experiments were performed for relatively short durations at certain temperatures. In real-world conditions, samples may be stored for extended periods of time at −20°C before processing, thus it may be valuable to assess ASFV DNA degradation under longer storage conditions.

Nevertheless, the data provided in this study are highly valuable to provide a baseline understanding of handling of environmental samples for the detection of ASFV DNA which can be used to establish the appropriate biosecurity procedures for ASFV preparedness and response.

## Data Availability

The original contributions presented in the study are included in the article/supplementary material; further inquiries can be directed to the corresponding authors.
